# Extracellular Vesicles: An Emerging Regenerative Treatment for Oral Disease

**DOI:** 10.3389/fcell.2021.669011

**Published:** 2021-05-17

**Authors:** Fanzhen He, Lu Li, Ruyi Fan, Xiaoqian Wang, Xu Chen, Yan Xu

**Affiliations:** ^1^Department of Periodontics, The Affiliated Stomatological Hospital of Nanjing Medical University, Nanjing Medical University, Nanjing, China; ^2^Jiangsu Province Key Laboratory of Oral Diseases, Nanjing Medical University, Nanjing, China; ^3^Jiangsu Province Engineering Research Center of Stomatological Translational Medicine, Nanjing Medical University, Nanjing, China

**Keywords:** dental pulp, periodontal tissue, cartilage, bone, oral tissue regeneration, extracellular vesicles

## Abstract

Extracellular Vesicles (EVs) are small lipid-enclosed particles containing biological molecules such as RNA and proteins that have emerged as vital modulators of intercellular communication. Increasingly, studies have shown that EVs play an essential role in the occurrence and prognosis of oral diseases. EVs are increasingly considered a research hotspot of oral diseases. In addition, the characteristics of carrying active molecules have also been studied in oral tissue regeneration. Evidence has shown that EVs regulate the homeostasis of the inflammatory microenvironment, promote angiogenesis, and repair damaged tissues. In this review, we summarized the characteristics of EVs and highlighted the role of EVs in oral tissue regeneration, including dental pulp, periodontal tissue, cartilage, and bone. We also discussed their deficiencies and prospects as a potential therapeutic role in the regeneration treatment of oral disease.

## Introduction

Extracellular vesicles (EVs) were reckoned as “platelet dust” in plasma when they were first observed by Wolf 50 years ago (Wolf, 1967). Since then, EVs have been found in many vivo biological fluids, including plasma, saliva, urine, breast milk, amniotic fluid, and cerebrospinal fluid (Jabbari et al., 2020b). Besides, it could also be found *in vitro* grown cell lines (Yáñez-Mó et al., 2015).

Extracellular vesicles are reservoirs of active molecules, including proteins, lipids, mRNA, ncRNAs, and DNA (Fatima et al., 2017). They provide a pivotal mean of communication and material transmission between neighboring and distant recipient cells in both physiological and pathological conditions (He et al., 2018; Kalluri and LeBleu, 2020). This ability makes them potentially valuable for scientific studies and clinical applications.

Evidence has indicated that EVs participate in many diseases. Importantly, in regenerative medicine, EVs derived from oral mesenchymal stem cells (MSCs) have potential in the regeneration of oral tissue, such as dental pulp or periodontal tissues (Chew et al., 2019; Sjöqvist et al., 2019; Shi et al., 2020). In this review, we outline the characteristics of EVs. In addition, we summarize and underscore the potential of EVs in oral disease regeneration treatment.

## Extracellular Vesicles

### Biogenesis

**FIGURE 1:**
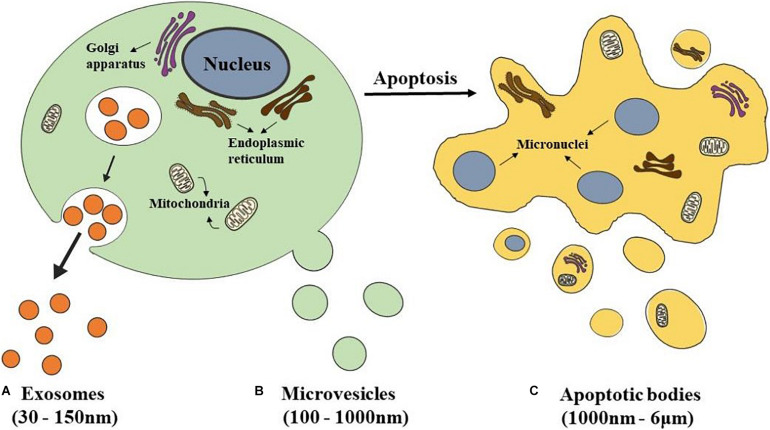
Schematics representation of different types of EVs. **(A)** Exosomes are released from eukaryotic cells by fusion of internal multivesicular compartments (MVB). **(B)** Microvesicles are formed by direct budding from the plasma membrane. **(C)** Apoptotic bodies are released from apoptotic cells.

According to size, biogenesis, and biochemical composition, there are three main classification EVs, including exosomes, microvesicles (MVs), and apoptotic bodies (Théry et al., 2018; Figure 1).

The most popular subclass of EVs is exosomes which have been studied the most by researchers. Exosomes have a double-layer lipid structure with a cup-shaped morphology and have a size range between 30 and 150 nm in diameter (Akbari et al., 2020; Figure 1A). They sprout from the organelle membrane to the cytoplasm. First, endosomes produced from endocytosis of the cell membrane cause multiple inward sprouts and form intraluminal vesicles (ILVs). Then ILVs transform into multivesicular bodies (MVBs) with dynamic subcellular structures, that is, late endosomes (Hanson and Cashikar, 2012). MVBs are generated at the endosome through two different processes. One is the endosomal sorting complex required for transport (ESCRT), and the other is the independent ESCRT mechanism (Gatta and Carlton, 2019; Vietri et al., 2020). The ESCRT mechanism is controlled by a group of cytoplasmic protein complexes that recognize ubiquitin-modified membrane proteins. The ubiquitin marker is recognized by ESCRT-0. ESCRT-0 is collected into the endosomal membrane and transferred ubiquitin-modified molecules to ESCRT I and ESCRT II. ESCRTI recognizes the disulfide bond to induce indentation of the endosomal membrane and then cuts the bud neck-through ESCRT III to form MVBs (Alenquer and Amorim, 2015; Nikfarjam et al., 2020). MVBs fuse with the plasma membrane and are secreted extracellularly, forming exosomes (de Souza and Barrias, 2020).

Microvesicles, which size range from 100–1,000 nm, are formed by direct budding from the plasma membrane (Nieuwland et al., 2018; Figure 1B). Their biogenesis occurs *via* the direct outward blebbing and pinching of the plasma membrane, releasing the nascent microvesicles into the extracellular space (Tricarico et al., 2017). MVs accommodate heterogeneous adhesion molecules, including integrins. When adhesion molecules are released by stem cells at different phases, they could manipulate vesicles’ transporting and incorporating. As a result, protein and lipids components of the plasma membrane would undergo significant, localized changes, leading to modulations in the curvature and rigidity of the membrane. Changes in plasma membrane also dominate the redistribution of the cargo contents, which are selectively enriched inside MVs (Zaborowski et al., 2015). Compared with the exosomes which are formed within MVBs, the process of MVs production represents a unique extracellular mechanism. This unique mechanism prompts the modified discharging of MVs carrying specially enriched molecular cargoes.

Apoptotic bodies, a category of EVs 1,000 nm–6 μm in diameter, are released from apoptotic cells (Ahmadi and Rezaie, 2020; Figure 1C). At the final stage of apoptosis, cells are disassembled into subcellular fragments, which turn into a variable number of apoptotic bodies. As a peculiar subclass of EVs, which may be more substantial than exosomes or MVs under certain conditions, and differ in composition, size, and structure (Jiang and Poon, 2019; Xu et al., 2019). The cellular components are various, including DNA fragments, micronuclei, chromatin residues, cytoplasmic parts, degraded proteins, and even intact organelles (Díaz-Flores et al., 2018). Current research has only shown that the formation of apoptotic bodies is the result of cell decomposition, which is a complex process involving highly coordinated morphological steps and biological processes. But the reason why different decomposition shows in various types of cells and the functional significance of this diversity remains unknown.

### Transmission

Extracellular vesicles play an essential role in intercellular communication and affect various cellular functions, including cytokine production, cell proliferation, apoptosis, and metabolism. Multiple studies have shown that EVs bind to the receptors of cells in three main ways (Guo et al., 2016; Jabbari et al., 2020a). First, EVs membranes and receptor cell membranes are directly fused. Second, EVs transfer membrane proteins or their contents to receptor cells and are completely swallowed by the receptor cell membrane. Third, the transmembrane protein on the EVs directly acts on the signal molecules on the surface of the receptor cell membrane. For example, EVs secreted by follicular dendritic cells carry major histocompatibility complex transmembrane proteins, which activate signaling molecules on the surface of B and T lymphocytes (Zhang and Yu, 2019). The way in which EVs bind to receptor cells depends on the size of the EVs and their contents. After EVs integrate with the target cell, the biological components could mediate intercellular signal transmission and play a key role in maintaining balance in the microenvironment and regulating physiological and pathological processes (Jia et al., 2017; Xu et al., 2018).

## EVs in Dental Pulp Regeneration

Dental caries can cause irreversible injury to dental pulp tissue, trauma, periodontitis, retrograde infection, et al. The primary treatment is root canal therapy, but some shortcomings include decreased tooth resistance and easy breakage (Tomson and Simon, 2016). Therefore, maintaining the pulp is the key to maintaining the function of teeth. Regeneration therapy replaces part of the damaged dental pulp tissue or allows pulp-like tissue to replace the original tissue completely (Huang et al., 2016).

Huang et al. (2016) cultured dental pulp stem cells (DPSCs) in standard medium and osteogenic induction medium for 4 weeks and then extracted EVs. It was found that EVs were endocytosed by DPSCs and human bone marrow mesenchymal stem cells (BMMSCs) in a dose-dependent manner. Both could activate the mitogen protein kinase (MAPK) pathway and promote osteogenic differentiation. The effect was better with the EVs extracted from the osteogenic induction medium than those from the standard medium. Then, they placed the EVs and collagen membrane on a 3–4 mm long dentin grinding piece and implanted it subcutaneously on the backs of nude mice. The immunohistochemical results showed that dentin matrix protein 1 (DMP1) and dentin sialophosphoprotein (DSPP) could be detected at the contact between the inner surface and soft tissue in both experimental groups. The results also showed that both EVs increased the expression of growth factors in the regenerated pulp-like tissues, such as bone morphogenetic protein-2 (BMP-2) and transforming growth factor-β (TGF-β). Additionally, increased proangiogenic factors and osteogenic differentiation factors, such as platelet-derived factor (PDGF) and Runt domain-related transcription factor 2 (RUNX2), were observed. The study above showed that EVs caused pulp-like tissue regeneration, and EVs that isolated under odontogenic conditions could induce stem cell differentiation and tissue regeneration with significant effects.

In previous studies, EVs extracted from the supernatant of DPSCs cultured in standard medium or under odontogenic differentiation conditions were named UN-EVs and OD-EVs, respectively. Twenty-eight microRNAs in OD-EVs were found to be changed by ion Torrent/MiSeq sequencing (Hu et al., 2019). Then, they discovered that UN-EVs only increased the expression of DSP in DPSCs. However, the endocytosis of OD-EVs triggered the odontogenic differentiation of DPSCs by upregulating DSP, RUNX2, DMP-1, and alkaline phosphatase (ALP). Further studies showed that microRNAs in EVs activated the TGF-β1 pathway in DPSCs by upregulating TGF-β1, transforming growth factor receptor 1 (TGFR1), pSmad2/3, and Smad4. It was suggested that EVs could be used as a biological tool for dental pulp regeneration, but they do not involve the recovery and regeneration of dental pulp nerves. Xian et al. (2018) revealed that the EVs of DPSCs enhanced vascular endothelial cells’ proliferation and angiogenic ability through the p38 MAPK pathway. Angiogenesis is an indispensable process for the survival of dental pulp upon infection or transplantation. This angiogenesis ability showed that EVs have great potential in dental pulp regeneration. Li et al. (2017) revealed that extracellular vesicles (SC-EVs) regulated the development of DPSCs. SC-EVs could significantly promote the proliferation and multidirectional differentiation of DPSCs. Although this was only for cells *in vitro*, it also provides a new strategy for studying dental pulp tissue regeneration (Yu et al., 2020).

**FIGURE 2:**
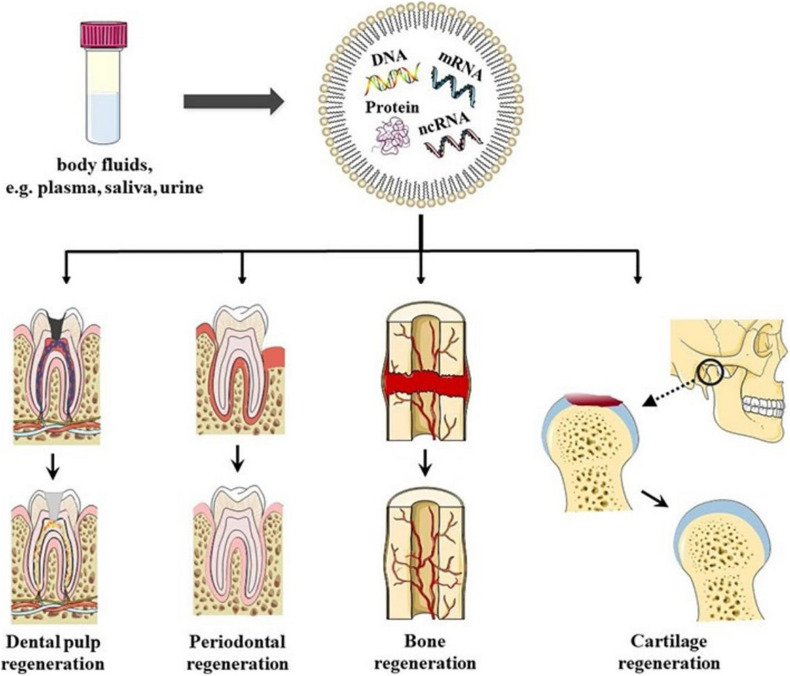
Schematics representation of EVs in oral tissue regeneration. EVs could be found in bodily fluids, for example, plasma, saliva, and urine. EVs have been shown to have the potential to induce dental pulp regeneration, periodontal tissue regeneration, Cartilage regeneration, angiogenesis, and bone regeneration, which provides new possibilities for regeneration therapy in oral disease in the future.

In conclusion, EVs have the potential to be used as biomimetic tools to induce DPSC differentiation. Furthermore, the sources and states of EVs donor cells play important roles in stem cell differentiation and tissue regeneration therapy (Figure 2).

## EVs in Periodontal Regeneration

Periodontal ligament cells (PLCs) belong to a heterogeneous cell group (Trubiani et al., 2019). Except for most fibroblasts, some cells express mesenchymal stem cell markers (such as Stro-1 and CD146), which are called periodontal ligament stem cells (PLSCs), differentiate into osteoblasts and cementoblasts. They also have the potential for multidirectional differentiation, which is an important basis for the regeneration of periodontal tissues (Seo et al., 2004).

Zhu et al. (Kolios and Moodley, 2013) extracted EVs from human BMMSCs and cocultured them with PLSCs. The study showed that these EVs promoted the proliferation and osteogenic differentiation of PLSCs *in vitro*. Researchers have confirmed that EVs derived from PLSCs carry a large around of *KAT6A* mRNA (Mens and Ghanbari, 2018). The osteogenic differentiation of PLSCs was promoted by upregulating the expression of KAT6A and affecting the level of histone acetylation. Wu et al. (Daley, 2015) established a periodontal defect rat model. EVs derived from deciduous dental pulp stem cells induced alveolar bone regeneration and angiogenesis. PLCs were cultured in a rat BMMSC-conditioned medium, which promoted the expression of osteogenesis-related genes and proteins, such as RUNX2, osteopontin (OPN), and osteocalcin (OCN), in PLCs (Shyh-Chang and Ng, 2017). The gel made with the same medium significantly increased the amount of alveolar bone in periodontal defect rats. EVs can be extracted from the cell culture medium and carry a variety of signaling molecules. Therefore, the abundant EVs in that study may be an essential factor for repairing alveolar bone in rats.

Researchers injected EVs derived from adipose stem cells (ASCs) into the periodontal pocket of rats induced by ligation (Mohammed et al., 2018). Histological examination showed that similar tissues formed in the defect area. Orderly periodontal ligament fibers attached to the cementum at one end and alveolar bone at the other end, similar to healthy periodontal tissue, were observed. Evidence has demonstrated that both ASCs and EVs have specific anti-inflammatory and immunomodulatory activities and induce tissue regeneration by promoting the migration, differentiation, and proliferation of different kinds of cells or angiogenesis. They also observed that EVs derived from ASCs had a better therapeutic effect on ligation-induced periodontitis than ASCs themselves, which showed a larger area of newly formed tissue (Mohammed et al., 2018).

Chew et al. (2019) loaded MSC-derived EVs into a collagen sponge. They placed it in the periodontal defect of rats, and the regeneration of alveolar bone and functional periodontal ligament fibers was observed. Studies have confirmed that EVs promote PLCs’ migration and proliferation *via* CD73-mediated adenosine receptor-activated AKT and extracellular regulated protein kinase (ERK) signaling pathways. *In vitro*, EVs were rapidly absorbed by PLCs after culturing for only a few minutes. In 15 min, signal transduction was induced. The expression of related genes, such as cell migration [insulin-like growth factor-1 (IGF-1) and fibroblast growth factor-2 (FGF-2)], survival, antiapoptosis [IGF-1, B-cell chronic lymphocytic leukemia/lymphoma-2 (BCL-2) and survival protein], cell proliferation (IGF-1, FGF-2 and proliferating cell nuclear antigen, proliferation nuclear antigen) and periodontal ligament matrix formation (extracellular matrix protein, collagen, and periosteal protein) genes, was continuously promoted. In addition, IGF-1 also promoted the osteogenic differentiation of PLCs and played an important role in the matrix mineralization of bone tissue. As an extracellular matrix protein, periosteal protein supports the adhesion and migration of fibroblasts and osteoblasts. Then, new bone was formed, and periodontal ligament attachment occurred by recruiting periodontal ligament fibroblasts and osteoblasts.

All these studies indicated that EVs have great potential in periodontal tissue regeneration, which also provides new possibilities for regeneration therapy for periodontal disease in the future (Figure 2).

## EVs in Cartilage Regeneration

Due to the limited self-healing ability of articular cartilage, temporomandibular joint osteoarthritis (TMJOA) is one of the most complex joint diseases to cure (Liu et al., 2020). The primary treatment strategy is preventing the gradual destruction of cartilage and subchondral bone, relieving pain, inducing regeneration, and restoring function (Wang et al., 2015). MSC-derived EVs have the ability to regulate the activity of immune effector cells and have the potential to induce MSCs’ multidirectional differentiation. The effect of EVs on cartilage regeneration has emerged in studies of the treatment of TMJOA (Luo et al., 2019).

It has been reported that MSC-derived EVs are carriers for intercellular communication (Dong et al., 2019). They transmitted the response of MSCs effectively to maintain the dynamic balance of the microenvironment in arthritis. Besides, MSC-derived EVs are rich in proteins and enzymes (Harrell et al., 2019). When the dynamic balance is disrupted due to injury and disease, enzyme-based EVs are activated. Activated EVs not only restore balance but also promote tissue function and induce tissue repair and regeneration. With the regeneration process, the activity of enzymes decreased (Lai et al., 2015). Based on these findings, EVs restored the dynamic balance of the microenvironment in the affected area during the occurrence of osteoarthritis. In addition, it induced the repair and regeneration of cartilage with endogenous signaling (Toh et al., 2017; Fang and Vangsness, 2020).

A study showed that in an immunoreactive TMJOA rat model, MSC-derived EVs played a vital role in regulating the inflammatory response. The repair of condylar cartilage and subchondral bone relieves pain (Zhang S. et al., 2019; D’Agnelli et al., 2020). In an immunoreactive rat model, MSC-derived EVs effectively repaired critical size cartilage defects (Zhang et al., 2018). In particular, MSC-derived EVs achieved therapeutic effects in TMJOA, and EVs mediated TMJOA recovery by regulating the inflammatory response (Kim et al., 2019). It has been reported that MSC-derived EVs promote the synthesis of a matrix composed of type II collagen and sulfated glycosaminoglycan (S-GAG) and accelerate the filling of tumor tissue. One group of mice was injected with EVs in the cartilage defect area. However, the control group was only injected with saline solution in the same area. Mice in the first group showed cartilage and subchondral bone regeneration, almost entirely recovering. In contrast, in the control group, the defect was only repaired by fiber, and the cartilage could not be regenerated (Zhang S. et al., 2016). This suggested that recovery can be achieved by injecting a therapeutic agent containing MSC-derived EVs into the damaged cartilage area.

Researchers found that MSC-derived EVs also induced cell proliferation and tissue regeneration *via* adenosine-mediated kinase phosphorylation. Adenosine activates AKT, ERK, and AMPK signals bind to EVs-mediated repair, thereby increasing chondrocyte activity. MSC-derived EVs promoted s-GAG synthesis. When s-GAG synthesis was inhibited by IL-1β, EVs inhibited the production of nitric oxide and matrix metalloproteinase 13 induced by IL-1β. The phosphorylation of AKT, ERK, and AMPK and the activation of adenosine receptors partially inhibited the effect of MSC-derived EVs (Johnson et al., 2012; Shah et al., 2018).

Previous studies have shown that MSC-derived EVs restore the matrix layer and overall homeostasis through several cellular processes (Mianehsaz et al., 2019). This result indicated that EVs-based therapy could be used as a cell-free method to treat temporomandibular joint pain and degeneration. EVs induce intercellular communication by transporting biological cargo in TMJOA (Asghar et al., 2020). This method induces cartilage and bone regeneration safely and effectively and reduces the intensity of pain, and restores mandibular function. Therefore, MSC-derived EVs may be a potential therapy for TMJOA recovery and regeneration (Figure 2).

## EVs in Bone Regeneration

Bone reconstruction is the key to maintaining the balance of bone metabolism. The bone-related cells that are involved are BMMSCs, osteoblasts (OBs), osteoclasts (OCs), and bone cells (Hadjidakis and Androulakis, 2006). Studies have shown that EVs promote the accumulation and osteogenic differentiation of host BMMSCs in damaged areas (Liu et al., 2018). Activation of OBs and OCs induces angiogenesis and bone regeneration (Qin et al., 2016; Zhu et al., 2019; Figure 2).

### Regulates the Osteogenic Differentiation of BMMSCs

Extracellular vesicles are transported between BMMSCs to regulate the differentiation process (Ren R. et al., 2019). BMMSCs and EVs secreted from human-induced pluripotent stem cell-derived mesenchymal stem cells (hiPSC-MSCs) were cocultured (Zhang J. et al., 2016). hiPSC-MSCs EVs entering BMMSCs were observed. Then, the proliferation and osteogenic differentiation of BMMSCs were induced. When a phosphoinositide 3-kinase (PI3K) inhibitor was added to the medium, the osteogenic process was inhibited. It was indicated that the promotion of proliferation and differentiation of BMMSCs by hiPSC-MSCs EVs was partly dependent on the PI3K/Akt pathway activation.

MiRNAs in EVs also play an essential role in the osteogenic differentiation of BMMSCs. Researchers found that miRNA inhibitors significantly attenuated OBs differentiation mediated by BMMSC-derived EVs (Lu et al., 2017). Studies have also demonstrated that miR-196a, miR-27a, and miR-206 carried by BMMSC-derived EVs play a major role in osteogenic differentiation. Liu et al. (2015) found that BMMSC-derived EVs transferred Fas protein to recipient cells, promoted recipient cell release of miR-29b, and enhanced the osteogenic differentiation ability of BMMSCs. EVs from BMMSCs in wild-type mice were transmitted to BMMSCs in abnormal osteogenic mice. Osteogenic differentiation was promoted by the increased expression of miR-151-5P in recipient cells (Chen et al., 2017).

In addition, EVs induce BMMSCs to secrete active factors required for bone repair and regeneration, which facilitates bone differentiation (Wang et al., 2019). Transwell results revealed that DC-derived EVs promoted the migration and recruitment of BMMSCs. Various recruitment factors, including OPN, matrix metalloproteinase-9 (MMP-9), and interleukin-5 (IL-5), have been detected in EVs (Silva et al., 2017). After coculture of DC-derived EVs and BMMSCs, the expression of osteoblastic proteins such as ALP and RUNX2 was significantly increased, and BMMSCs showed osteogenic differentiation (Fan et al., 2018).

### Regulation of the Proliferation and Activity of Osteoblasts and Osteoclasts

The growth, development, and regeneration of bone are closely related to OBs (Chang et al., 2019). OBs are differentiated from BMMSCs and have a prominent role in bone formation, reconstruction, and renewal. The function of OBs is regulated by hormones, proteins, miRNAs, et al. (Wei and Karsenty, 2015; Komori, 2019; Narayanan et al., 2019). EVs induce bone regeneration by regulating the proliferation and differentiation of OBs.

Bone marrow mesenchymal stem cells transmit miR-196a to osteoblasts through EVs to promote the expression of osteogenic genes. When miR-196a inhibitors were added to EVs, the expression of osteogenic genes in osteoblasts was significantly downregulated (Qin et al., 2016). In osteonecrosis, BMMSCs transfer miR-122-5p to OBs through EVs, and proliferation is induced (Liao et al., 2019). EVs also carry miR-122-5p to activate the MAPK pathway and receptor tyrosine kinase to facilitate the proliferation and differentiation of OBs and induce bone regeneration in osteoporosis (Liao et al., 2019). BMMSC-derived EVs enhanced the expression of osteogenic genes and activated alkaline phosphatase in OBs. Therefore, it promoted bone recovery of skull defects in rats (Qi et al., 2016). In addition, studies have found that EVs derived from prostate cancer cells increased the proliferative activity of OBs by 1.5 times. EVs activate ALP and induce OBs to produce more calcium deposits (Inder et al., 2014). It was reported that OB-derived EVs also have the potential for bone regeneration.

After bone marrow stromal cells phagocytose mineralized osteoblast EVs, mineralized OBs EVs target the *Axin1* gene through miR-667-3p, miR-6769b-5p, and activate the Wnt signaling pathway, leading to bone marrow stromal cells differentiating into OBs and enhancing osteogenesis (Cui et al., 2016). Mature OBs can activate the VEGF/ERK1/2 signaling pathway of endothelial cells, enhance endothelial cell proliferation, migration, and angiogenesis, and indirectly promote bone regeneration (Tang et al., 2019).

Other studies have shown that OBs promote the formation of OCs by secreting EVs that carry receptor activator of nuclear factor-κ B ligand (RANKL) (Chen et al., 2018). Bone metabolism mainly depends on the interaction between the osteogenic differentiation of OBs and the bone-breaking function of OCs (Luby et al., 2019). Therefore, regulating the function of OCs is also crucial in osteogenesis. EVs derived from prostate cancer cells inhibited the differentiation of OCs by downregulating miR-214 and blocked NF-κB signal transduction (Duan et al., 2019). It also regulated bone formation by reducing the expression of osteoclast markers such as dendritic cell-specific transmembrane protein (DC-STAMP), tartrate-resistant acid phosphatase (TRAP), cathepsin K and MMP-9, which inhibited the proliferation and differentiation of OCs (Karlsson et al., 2016). ASC-derived EVs not only reduced the mRNA and protein levels of RANKL but also diminished the RANKL/OPG ratio; thus, RANKL-mediated OCs differentiation was inhibited (Ren L. et al., 2019). After injection of EVs derived from endothelial cells in estrogen-deficient osteoporotic model mice, the activity of OCs decreased and effectively inhibited the development of osteoporosis (Song et al., 2019).

### Regulate Angiogenesis

Bone is a highly vascularized tissue that relies on the close relationship between blood vessels and osteocytes to maintain integrity (Takeuchi et al., 2019). Therefore, angiogenesis plays an essential physiological role in the process of bone regeneration. Neovascularization not only transports oxygen and nutrients but also promotes cell proliferation and bone matrix mineralization (Teng et al., 2015).

Academics have found that EVs derived from hamsas promote angiogenesis *in vitro* (Zhu et al., 2018). Further studies showed that the miRNAs in EVs, such as miR-126, miR-130a, and miR-132, played an important role in regulating vascular endothelial cell proliferation and angiogenesis (Liu et al., 2017). hiPSC-derived EVs promoted the proliferation, migration, and angiogenesis of vascular endothelial cells by activating the P13-resistant Akt pathway. The EVs of endothelial progenitor cells promote the proliferation, migration, and angiogenesis of endothelial cells through the transmission of miR-126. The blood vessel density around the tibia of rats was increased, thus effectively accelerating bone regeneration in the defective area (Jia et al., 2019). Researchers injected BMMSC-derived EVs into rats with a femoral fracture. Then, EVs promoted endothelial cell proliferation, migration, lumen formation, and angiogenesis and facilitated fracture recovery. Further studies have shown that EVs promote the expression of vascular endothelial growth factor (VEGF) by inducing HIF-1α and induce bone regeneration (Zhang Y. et al., 2019; Ying et al., 2020).

It has been reported that under chronic hypoxia, miR-135b is highly expressed in EVs, which are secreted by multiple myeloma cells. miR-135b accelerated angiogenesis by targeting the FIH-1/HIF-1 pathway in endothelial cells (Umezu et al., 2014). In addition to hypoxia, biomaterials also promote angiogenesis. Studies combined rat BMMSC-derived EVs with tissue engineering scaffolds *in vivo* (Xie et al., 2017). The results showed that bone and vascular regeneration were effectively promoted. Another biomaterial that contained lithium-induced the expression of miR-130a in EVs activated the PTEN/AKT pathway and then promoted angiogenesis in bone regeneration (Liu et al., 2019).

## Conclusion and Perspective

Previous studies have proven that EVs can be separated from various body fluids and are quite stable at −80°C for long-term preservation (Xu et al., 2014; Wu et al., 2019). Additionally, EVs can surround bioactive substances and protect them from being degraded by enzymes (Shimoda and Khokha, 2017). In addition, EVs can be further modified to meet the needs of specific treatment options (Huang et al., 2019; Shimoda, 2019). These characteristics indicate the feasibility of EVs-based treatment.

However, there are many limitations and challenges in the clinical application of EVs in regeneration treatment. First, an effective way to separate and purify EVs from cells or fluids in large quantities has not been found. Second, transportation and storage conditions still need further study. At present, EVs are extracted mainly by ultra-speed centrifugation, immunoadsorption, precipitation, or microfluid separation (Koritzinsky et al., 2017). The yield of EVs isolated *in vitro* is low, and EVs are easily contaminated by proteins or other extracellular vesicles. Factors such as storage temperature, storage solution pH, or freeze-thaw cycle could also affect the activity of EVs (Pegtel and Gould, 2019).

Additionally, the mechanism of EVs that carry a variety of bioactive molecules and communicate between cells is not precise (Lin et al., 2015). There is a certain risk if it cannot be accurately regulated. Investigations have found that BMMSC-derived EVs promote tumor occurrence and development by transmitting related miRNAs to tumor cells or activating signaling pathways (Sharma, 2018; Wang et al., 2020). The mechanism of tissue repair and regeneration has not been fully elucidated either. It is unclear exactly how the amount of EVs used for treatment is measured and whether excessive EVs may cause irreversible tissue damage. Therefore, if we want to apply EVs-based regeneration therapy, it is necessary to clarify its transport path, dose, biological distribution, and metabolic kinetics. Only in this way can EVs be functional and ensure the safety of their clinical application.

In conclusion, although challenges and difficulties remain, EVs still show great potential in biomedicine. EVs-based treatment remains a promising approach, and EVs combined with tissue engineering can also provide new tissue regeneration ideas. It has important research significance and broad application prospects in the future.

## Author Contributions

All authors contributed equally to the article and approved the submitted version.

## Conflict of Interest

The authors declare that the research was conducted in the absence of any commercial or financial relationships that could be construed as a potential conflict of interest.
